# Supplementary material for: The G119S Acetylcholinesterase (Ace-1) Target Site Mutation Confers Carbamate Resistance in the Major Malaria Vector Anopheles gambiae from Cameroon: A Challenge for the Coming IRS Implementation
Source: Genes (Basel). 2019 Oct 11;10(10):790. doi: 10.3390/genes10100790 (PMC6826778; doi:10.3390/genes10100790)
Supplement: Supplementary file 1 [file genes-10-00790-s001.zip › File S1.docx]

**Additional file 1** : Raw counts of individuals for each ace-1 genotype 24h after exposure to bendicarb (table 1) and propoxur (table2)

**Table 1**: Number of individual for each genotype according to insecticide profile after exposure to bendiocarb

| Genotypes | Number of Alive mosquitoes (frequency) | Number of dead mosquitoes (frequency) | total |
| --- | --- | --- | --- |
| S/S | 19 (76%) | 3 (4.5%) | 22 |
| G/S | 2 (8%) | 1 (1.5%) | 3 |
| G/G | 4 (16%) | 63 (94%) | 67 |
| Total | 25 | 67 | 102 |

**Table 2**: Number of individual for each genotype according to insecticide profile after exposure to propoxur

| Genotypes | Number of Alive mosquitoes (frequency) | Number of dead mosquitoes (frequency) | total |
| --- | --- | --- | --- |
| S/S | 29 (97%) | 0 | 29 |
| G/S | 0 | 0 | 0 |
| G/G | 1 (3%) | 38 (100%) | 39 |
| Total | 30 | 38 | 68 |
