# Supplementary material for: The G119S Acetylcholinesterase (Ace-1) Target Site Mutation Confers Carbamate Resistance in the Major Malaria Vector Anopheles gambiae from Cameroon: A Challenge for the Coming IRS Implementation
Source: Genes (Basel). 2019 Oct 11;10(10):790. doi: 10.3390/genes10100790 (PMC6826778; doi:10.3390/genes10100790)
Supplement: Supplementary file 1 [file genes-10-00790-s001.zip › File S4.docx]

**Additional file 4**: Summary statistics for polymorphism in a common region of (703bp) in Ace-1 gene for the directly sequenced and cloned samples including the G119S mutation in An. gambiae mosquito population from Bankeng,

|  | **2n** | **S** | **h** | **hd** | **π** | **D** | **D*** | **Fs** |
| --- | --- | --- | --- | --- | --- | --- | --- | --- |
| **Resistant** | 58 | 20 | 18 | 0.635 | 0.004 | -1.306ns | 0.875ns | 0.183ns |
| **Susceptible** | 41 | 35 | 21 | 0.927 | 0.009 | -1.113ns | -0.315ns | -5.627* |
| **Total** | 99 | 42 | 39 | 0.859 | 0.01 | -0.461ns | -1.049ns | -11.859* |

Central Cameroon
